# Gender Differential Expression of AR/miR-21 Signaling Axis and Its Protective Effect on Renal Ischemia-Reperfusion Injury

**DOI:** 10.3389/fcell.2022.861327

**Published:** 2022-04-28

**Authors:** Gaomin Huang, Qiu Yao, Zhenfeng Ye, Yawei Huang, Chiyu Zhang, Yi Jiang, Xiaoqing Xi

**Affiliations:** Department of Urology, Second Affiliated Hospital of Nanchang University, Nanchang, China

**Keywords:** gender difference, androgen receptor, miR-21, renal ischemia-reperfusion, PDCD4

## Abstract

**Objective:** The aim of this study was to investigate gender differences after renal ischemia-reperfusion injury in mice and the effects of androgen receptor (AR) and microRNA-21 (miR-21) on apoptosis in renal ischemia-reperfusion injury.

**Methods:** Renal ischemia-reperfusion injury model was induced by 45 min of bilateral renal artery ischemia and reperfusion. BALB/c mice were randomly divided into groups according to different experimental protocols. The levels of renal function were evaluated by serum creatinine and blood urea nitrogen. TUNEL staining was used to analyze the pathological changes and apoptosis levels of renal tissue, and western blotting and qPCR were used to detect the expressions of miR-21, AR, PDCD4 and caspase3.

**Results:** After renal ischemia-reperfusion injury in mice with different genders, the levels of plasma urea nitrogen and creatinine in female and male mice increased, the histopathological score increased, and TUNEL staining in renal tissue indicated increased apoptosis. The expressions of miR-21, PDCD4, and active caspase-3 protein were up-regulated. The above trend was more pronounced in male mice, and a significant decrease in AR mRNA expression was detected. Silencing the expression of AR aggravated the decline of renal function and renal tubular injury after renal ischemia in mice. The expression of PDCD4 and active caspase-3 increased, while the level of miR-21 was correspondingly decreased. Up-regulation of miR-21 expression by pre-miR-21 could negatively regulate PDCD4, reduce the expression level of active caspase3, and yet induce AR expression accordingly. MiR-21 alleviated renal ischemia-reperfusion injury by inhibiting renal tubular epithelial cell apoptosis. The effect of antagomiR-21 was the opposite, which aggravated renal ischemia-reperfusion injury.

**Conclusion:** There are gender differences in renal ischemia-reperfusion injury. Male mice are more susceptible to renal ischemia-reperfusion injury than female. Silencing AR expression or down-regulating the level of miR-21 can promote the expression of PDCD4 and apoptosis protein caspase3, thereby aggravating ischemia-reperfusion injury in mice. The protective effect of AR and miR-21 in renal ischemia-reperfusion injury has a certain synergy.

## Introduction

Renal ischemia-reperfusion (renal IR) injury is a common pathophysiological process in clinical practice and the main cause of acute renal failure (Murugan and Kellum, 2011). At present, the mechanism of renal IR is not fully recognized. It usually involves a variety of pathophysiological processes such as oxidative stress, inflammatory response, and apoptosis of renal tubular epithelial cells (Lee et al., 2019). In the early stage of renal ischemia, the interaction between damaged renal tubular epithelial cells, activated endothelial cells and macrophages induces oxidative stress and complement activation, aggravating the process of cell damage. Acute inflammation occurs after blood flow reperfusion, including the production of many pro-inflammatory factors, such as tumor necrosis factor-alpha (TNF-α), interleukin-1beta (IL-1β), and induces a large amount of apoptosis of renal tubular epithelial cells (Mulay et al., 2016). The pathophysiological mechanism of renal IR is definite complicated, and the molecular mechanism of IR-induced renal tubular cell death has not yet been fully elucidated. Therefore, the research on the protection strategy and pathogenesis of renal IR is of great significance.

In recent years, studies have found that in acute ischemic injury, there are gender differences between male and female individuals. According to reports, androgens protect male mice from ischemia-induced castration-related damage by regulating angiogenesis in cardiovascular diseases, and rely on the transcriptional activation of androgen receptors (Lam et al., 2016). Some scholars have found through animal experiments and clinical cases that there are obvious gender differences in the sensitivity and tolerance of the kidney to ischemia-reperfusion injury. The animals show that female rats are more resistant to ischemic kidney injury than male rats (Mehta et al., 2002; Kim et al., 2006; Kang et al., 2014), we also observed this phenomenon in the experiment of sex difference in mice. According to reports, acute ischemic diseases may reduce androgen levels (Wagner et al., 2011). Further studies by researchers have shown that renal ischemia-reperfusion injury leads to a decrease in androgen levels, and androgen supplementation can protect renal ischemic injury, and castration can promote renal injury and renal failure (Robert et al., 2011; Soljancic et al., 2013). The effects of androgens on target organs are mainly mediated by nuclear androgen receptor (AR) transcriptional control of target genes. Androgens also interact with a variety of signaling pathways independent of transcriptional control through AR in the cytoplasm, thereby inducing rapid activation of the kinase signaling cascade (Baron et al., 2004). In order to clarify the role of AR in the gender difference of renal IR, we detected the AR transcription level of the mouse kidney 24 h after IR in the mouse ischemia-reperfusion injury model, and found that the AR level in the kidney tissue of male mice decreased significantly. However, it is not clear whether AR plays a unique pathophysiological role in renal ischemia.

MicroRNAs (miRNAs) are a type of endogenous non-coding RNA found in eukaryotes, with a size of about 20 nucleotides in length. miRNAs inhibit gene expression after transcription by targeting the 3′UTR of mRNA, and participate in a variety of regulatory pathways, including immune response, metabolic processes, tumorigenesis and development, organ formation, cell proliferation, and apoptosis (Hwang and Mendell, 2006; Tavazoie et al., 2008; Lu et al., 2011; Rottiers and Näär, 2012; Lu and Rothenberg, 2018). More and more studies have found that microRNA can regulate IR-induced kidney damage by regulating the expression of target genes (Hao et al., 2017; Jia et al., 2020). Studies have shown that miR-21 participates in ischemia-reperfusion injury of a variety of tissues and organs, including the heart, kidney, intestine, and brain through the regulation of apoptosis, inflammation, and oxidative stress (He et al., 2016; Yang et al., 2016; Jia et al., 2017). We have previously reported that miR-21 can reduce renal ischemia-reperfusion injury by reducing the expression of its target gene PDCD4 and inhibiting apoptosis (Hu et al., 2014). Here, we found that the expression of miR-21 in kidney tissues of mice of different genders has obvious gender differences after renal IR. The mechanism of miR-21 involved in renal ischemia-reperfusion is still worth studying. In addition, some researchers recently found that AR can positively regulate the expression of miR-21 in prostate cancer, and there is a positive feedback loop between the regulation of AR and miR-21 (Mishra et al., 2014). However, whether miR-21 could interact with AR to participate in gender differences in renal IR injury remains unknown. In order to clarify this question, this study used a mouse ischemia-reperfusion injury model to explore the role of AR and miR-21 in renal ischemia-reperfusion injury.

## Materials and Methods

### Experiment Animals

A total of 90 male and female BALB/c mice (6–8 weeks old, weighing about 20–25 g) were purchased from the Animal Experimental Center of Nanchang University. Animals are kept in a 12/12 h light/dark cycle environment, where food and water are freely available. The experimental protocol in this study was approved by the Animal Care and Use Committee of Nanchang University.

### Renal Ischemia-Reperfusion Injury Model

Based on our previous work, mouse renal ischemia-reperfusion model was established. The mice were fasted for 8 h before the operation, injected with sodium pentobarbital (50–60 mg/kg, ip) into the abdominal cavity to induce anesthesia, fixed the limbs and disinfected with iodophor before the operation. The renal pedicles were exposed through an abdominal incision, and the bilateral renal arteries were clamped for 45 min. During reperfusion, loosen the hemostatic clip, observe the color change of the kidney, confirm that the blood flow is normal, and suture the incision. The sham operation group performed the same operation without clamping the renal artery. We used a heating pad to maintain the mouse’s body temperature at 35–37°C to ensure that the operation was performed at a constant temperature and promote the recovery of the mouse after surgery. The kidney was obtained 24 h after the operation and the serum was collected. Two weeks before surgery, the kidney was exposed through an incision in the subcostal renal region. Then 50 ul lentivirus constructs containing AR-shRNA (3*10E7 TU/ml, Genechem, Shanghai, China) was injected into the upper and lower poles of the renal, while equal concentration lentivirus of scrambled shRNA was used as negative control. Pre-miR-21 (RiboBio, Guangzhou, China) was used as an activator of miR-21 overexpression at a concentration of 5 mg/kg, and AntagomiR-21 (RiboBio, Guangzhou, China) was used as an inhibitor of miR-21 at 10 mg/kg. Activators and inhibitors were injected intraperitoneally 24 and 1 h before renal IR surgery.

### Grouping

According to the experimental protocol, the mice were divided into several groups, *n* ≥ 5 in each group. We tested the effects of IR on AR and miR-21 in renal tissues of different genders, and divided them into four groups: 1) male renal IR, 2) female renal IR, 3) male sham operation, and 4) female sham operation. We studied the effect of interfering with AR expression on IR injury and divided male mice into six groups: 1) AR-shRNA + IR, 2) AR-NC + IR, 3) Control + IR, 4) AR-shRNA + sham operation, 5) AR-NC + sham operation, and 6) Control + sham operation. We explored the role of miRNA in protecting renal IR injury. Male mice were randomized into six groups: 1) pre-miR-21 + IR, 2) AntagomiR-21 + IR, 3) Control + IR, 4) pre-miR-21 + sham operation, 5) AntagomiR-21 + sham operation, and 6) Control + sham operation.

### Serum Creatinine and Blood Urea Nitrogen Determination

Serum creatinine (Cr) and blood urea nitrogen (BUN) were measured using Cr determination kit and BUN determination kit (Rayto, Shenzhen, China). Collect blood samples from the animal’s tail or at the time of execution. After standing at room temperature for 2 h, the serum was collected by centrifugation. The samples were analyzed on a fully automatic biochemical analyzer (Rayto, Chemray 240, Shenzhen, China), and the corresponding parameters followed the manufacturer’s instructions.

### Histopathological Analysis

Kidney tissues were separated from mice and fixed with 4% paraformaldehyde, embedded in paraffin, and sliced at 4 um. Tissue sections were stained with hematoxylin and eosin (HE) using standard procedures. Observe the tissue sections under an optical microscope (Olympus, Tokyo, Japan), and score the pathology according to the percentage of necrosis or necrotic fragments in the proximal tubules of the cortex and outer medulla: 0, normal kidney; 1, <10%; 2, 10–25%; 3, 26–75%; 4, >75%.

### TUNEL Staining

TUNEL method was used to detect renal tissue apoptosis. According to the manufacturer’s instructions, use TUNEL detection kit (Servicebio, Wuhan, China) to detect 4 um tissue sections. First, the paraffin-embedded tissue sections were deparaffinized and transparent, and then the sections were repaired with proteinase K working solution for 30 min, and 0.1% triton was added dropwise to the sections and incubated at room temperature for 20 min. Then, the sections were incubated in a mixed buffer containing TDT enzyme-dUTP at 37°C for 2 h. DAPI staining solution was added dropwise and incubated at room temperature in the dark for 10 min. The sections were washed with PBS, and finally mounted with anti-fluorescence quenching mounting tablets, and the sections were observed through a fluorescence microscope to estimate the number of TUNEL-positive staining cells.

### Quantitative PCR

Total RNA was extracted from the tissues using TransZol Up kit (Transgen, Beijing, China). Use Easyscript kit with gDNA Remover (Transgen, Beijing, China) to reverse transcribe the extracted RNA into cDNA, and then use Green qPCR Supermix kit (Transgen) to perform qPCR in real-time fluorescence quantitative PCR Detection System (Applied Biosystems, 7900, United States). PCR primers are synthesized by Sangon (Shanghai, China), using to obtain the expression level of mRNA. The primers (5′-3′) used were listed as follows: AR (mice) forward primer: TCC​AAG​ACC​TAT​CGA​GGA​GCG, reverse primer: GTG​GGC​TTG​AGG​AGA​ACC​AT; PDCD4 (mice) forward primer: AAA​GAC​GAC​TGC​GGA​AAA​ATT​CA, reverse primer: CTT​CTA​ACC​GCT​TCA​CTT​CCA​TT; GAPDH (mice) forward primer: AGG​TCG​GTG​TGA​ACG​GAT​TTG, reverse primer: TGT​AGA​CCA​TGT​AGT​TGA​GGT​CA; U6 (mice) forward primer: CTCGCTTCGGCAGCACA, reverse primer: AAC​GCT​TCA​CGA​ATT​TGC​GT. For the detection of miR-21 expression, use the mmu-miR-21a-5p bulge-loop RT primer designed by RiboBio (Guangzhou, China) for reverse transcription, and then use the mmu-miR-21a-5p specific primer pair for qPCR. MiRNA uses U6 as an internal reference, and mRNA uses GADPH as an internal reference. Use the 2-ΔΔCt value to quantify the fold change and determine relative gene expression.

### Western Blot

The western blot analysis was performed according to the standard protocol. The tissue was lysed with RIPA buffer (Solarbio, Beijing, China) containing 1 mM PMSF. After high-speed centrifugation, use the loading buffer to collect the tissue lysate. An equal amount of protein samples is separated on a 10% SDS-PAGE gel and then transferred to a PVDF membrane. And sealed in 5% skimmed milk for 1 h. PDCD4 antibody, cleaved caspase-3 antibody, Caspase-3 antibody, and GAPDH antibody were diluted by 1:500, 1:1000, 1:1000, and 1:5000, respectively. The membrane was incubated with the primary antibody overnight at 4°C, and then incubated with the horseradish peroxidase (HRP) secondary antibody (1:10,000) for 1 h at room temperature, and finally the detection strip was incubated with ECL working solution (Us Everbright, Suzhou, China). Antibodies were purchased from the following sources: anti-PDCD4 (catalogue number: ab80590) from Abcam, anti-cleaved-caspase-3 (catalogue number: 9661) from Cell Signaling Technology, anti-caspase-3 (catalogue number: 66470-2-Ig) and anti-GAPDH (catalogue number: 60004-1-Ig) from Proteintech.

### Statistical Analysis

The data are expressed as mean ± standard deviation (SD) (*n* > 5), and the difference between the two groups was tested by t test. A two-way ANOVA multiple-comparisons test is used to estimate how two factors affect a response variable. *p* < 0.05 is considered to reflect a significant difference between the two sets of data.

## Results

### Effect of Sex Difference on Renal Function and Histological Changes After Renal Ischemia-Reperfusion Injury

**FIGURE 1:**
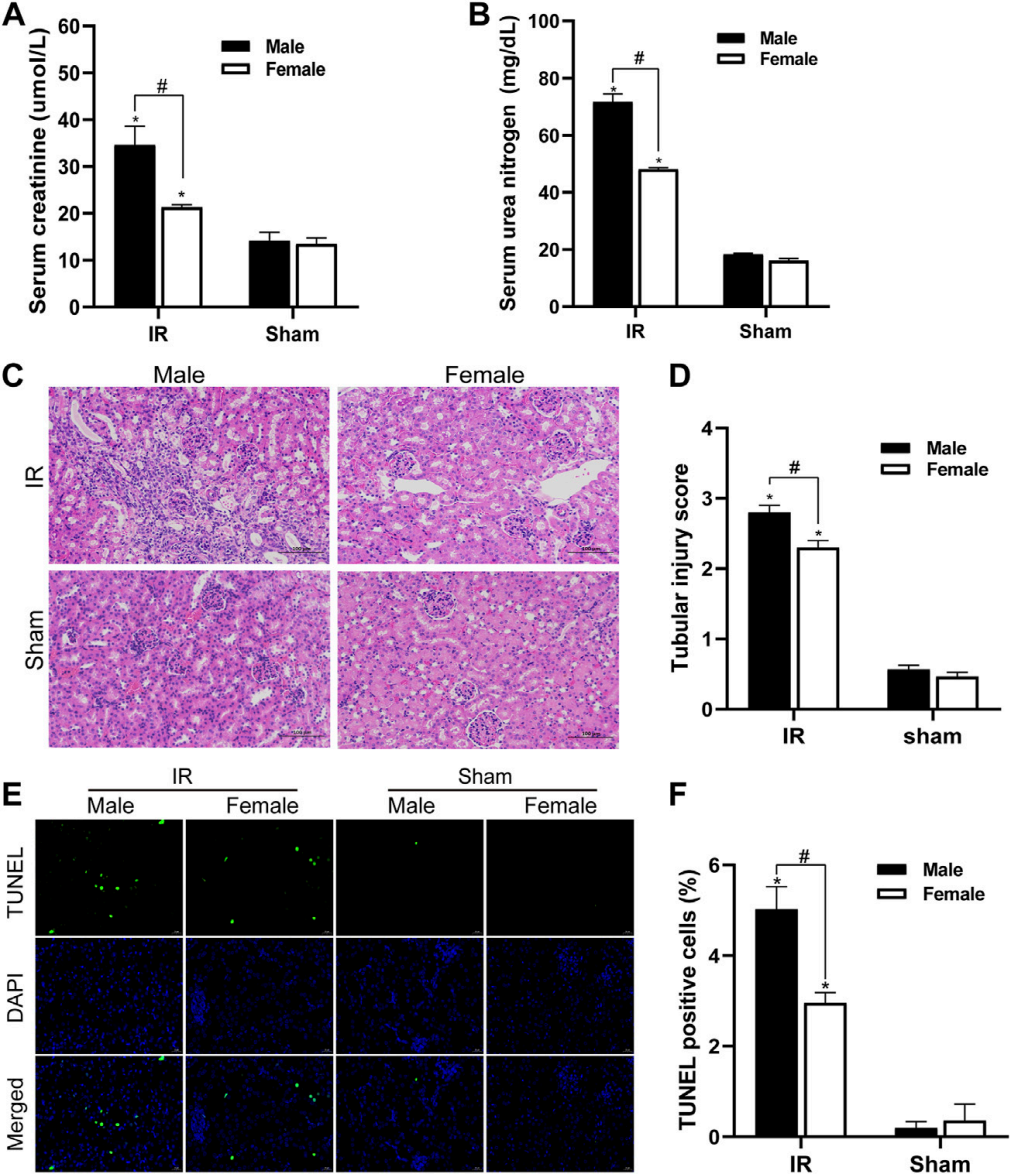
Gender differences in renal ischemia-reperfusion injury. **(A,B)** Serum creatinine levels **(A)** and serum urea nitrogen levels **(B)** of male and female mice in renal ischemia for 45 min and 24 h after reperfusion. **(C)** HE staining of renal tissue (original magnification ×200). Renal tubule injury is characterized by renal tubular atrophy and dilation, accompanied by tubular type. **(D)** Corresponding renal tissue pathological damage score. **(E)** Representative photograph for TUNEL staining section of renal tubular epithelial cells (green) in each group. Original magnification: ×400. Scale bar: 20 µm. **(F)** Quantitative evaluation of TUNEL-positive cells in renal tissue. The data displayed are mean ± SEM (*n* > 5). Compared with sham operation group, *p < 0.05. Compared with female renal IR group, #*p* < 0.05.

We detected the changes in serum creatinine and serum urea nitrogen levels in female and male mice after renal ischemia for 45 min and reperfusion for 24 h. The serum creatinine level of the control group animals was 12–16 umol/l, and the BUN value was about 17 mg/dl. There was no significant difference between male and female individuals. Compared with the control group, serum creatinine and serum urea nitrogen levels increased significantly after IR injury. However, compared with female mice, male mice had a more significant decline in renal function caused by IR injury (Figures 1A,B). The HE staining of kidney tissue is as shown in Figure 1C. There was no significant change in the kidney histology of mice in the control group. After ischemia-reperfusion, both male and female mice showed renal tubular injury, and many renal tubular atrophy and dilation were seen in the renal tissue, but the degree of injury in male animals was significantly higher. In order to quantify the kidney tissue damage in mice, the renal tissue pathological damage score was used to determine the percentage of renal tubular necrosis and dissolution. The results showed that the pathological score of the renal tissue of male mice was close to three and that of female mice was approximately 2. The pathological score increased after IR injury, but the increase in male mice was more obvious (Figure 1D). Tubular damage and necrosis under ischemic conditions are closely related to cell apoptosis. In order to study the effect of gender on renal tubular cell apoptosis after IR injury, the TUNEL method was used to detect renal tissue in our experiment. After ischemia-reperfusion, a large number of TUNEL-positive cells appeared in the kidney tissue of male mice, while TUNEL-stained cells were relatively few in female mice. TUNEL-positive cells were not found in the control group. TUNEL staining of renal tissues indicated that the renal cell apoptosis in the IR group was significantly increased, and the male renal cell apoptosis in the IR group was more severe than that of the female (Figures 1E,F).

### The Influence of Gender Differences on the Expression Levels of Androgen Receptor and miR-21 After Ischemia-Reperfusion injury

**FIGURE 2:**
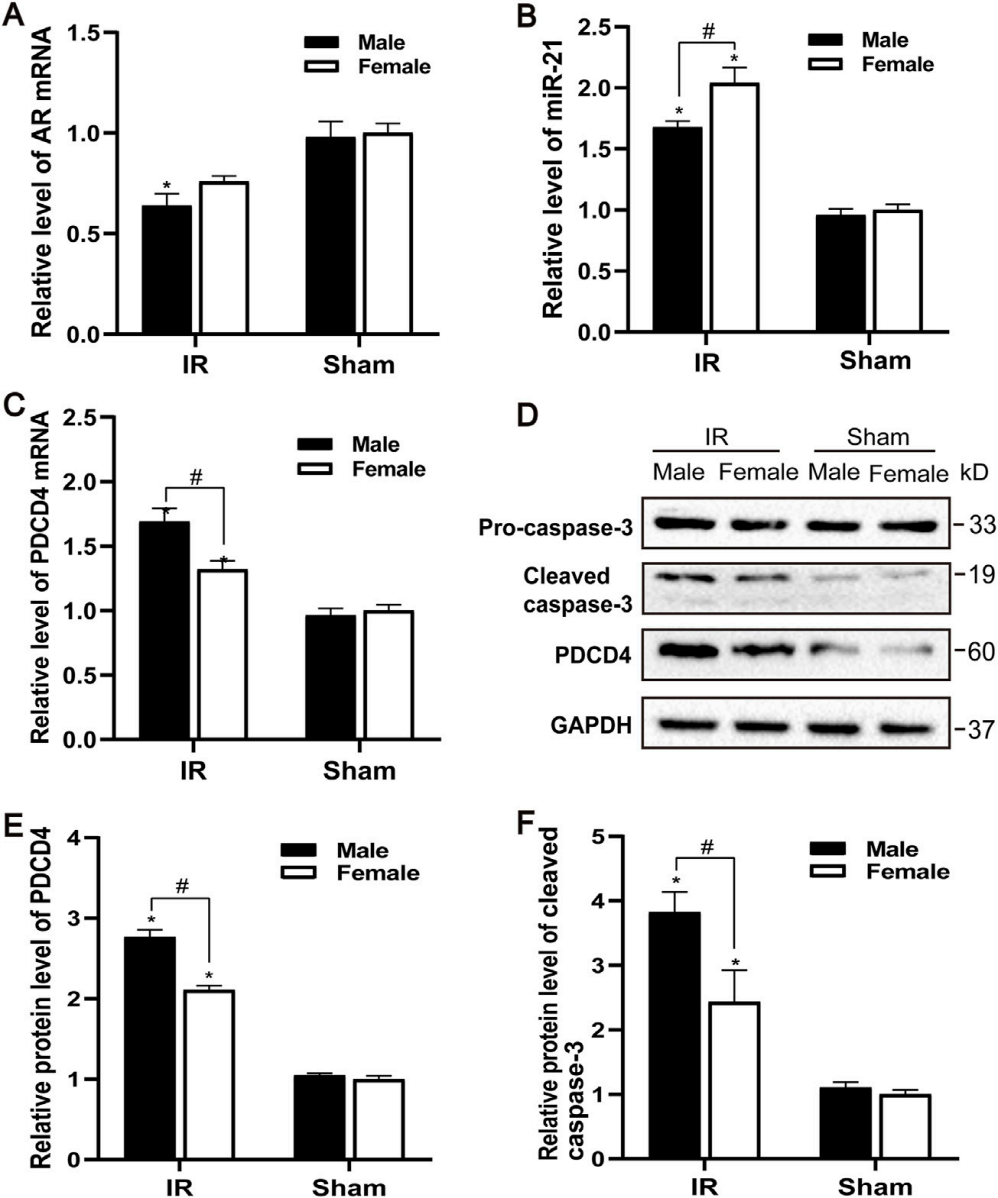
Gender difference expression of AR and miR-21 after renal IR injury. **(A–C)** qPCR showing AR **(A)**, miR-21**(B)**, and PDCD4 **(C)** mRNA levels of male and female mice in renal ischemia for 45 min and 24 h after reperfusion. **(D)** The expression of cleaved caspase-3 and PDCD4 protein in renal tissue after renal ischemia-reperfusion was detected by Western blot. **(E)** Quantitative evaluation of PDCD4 protein levels in renal tissue. **(F)** Quantitative evaluation of cleaved caspase-3 protein levels in renal tissue. Data presented are mean ± SEM (*n* > 5). Compared with sham operation group, *p < 0.05. Compared with female renal IR group, #*p* < 0.05.

The AR expression of male and female mice after IR injury was evaluated. Compared with the control group, the levels of AR mRNA in male mice decreased to varying degrees, and the decrease of AR in female mice was not significant (Figure 2A). Male mice may be more sensitive to AR decline than female mice, causing renal ischemia-reperfusion injury more severe than female mice. Compared with the sham group, the expression of PDCD4 mRNA and protein in the IR group was significantly increased, and male mice were more significant than female mice (Figures 2C–E). Compared with the control group, the expression of miR-21 was significantly increased after IR injury, while the expression of miR-21 between male and female mice was also significantly different. Compared with male mice, the expression of miR-21 in female mice risen more significantly (Figure 2B). Caspase-3 is an important part of the caspase-dependent apoptosis pathway. In the experiment, we used western blotting to detect the expression of caspase-3 protein in kidney tissue. Compared with the sham group, the expression of apoptotic factor caspase-3 was increased after IR injury, while the expression of caspase-3 in the renal tissue of male mice was significantly higher than that of female mice, which is consistent with the TUNEL results (Figures 2D,F).

### Silencing Androgen Receptor Expression Aggravates Renal Ischemia-Reperfusion Injury

**FIGURE 3:**
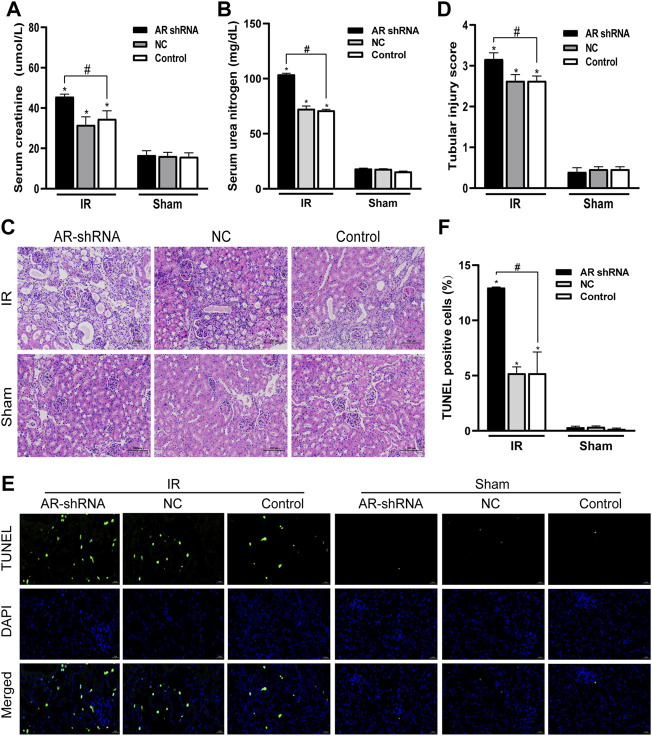
Silencing AR expression aggravates renal ischemia-reperfusion injury in male mice. **(A,B)** Effects of AR-shRNA silencing AR expression on the level of serum creatinine **(A)** and serum urea nitrogen **(B)** in male mice. **(C)** HE staining of renal tissue in each group (original magnification ×200). **(D)** Corresponding renal tissue pathological damage score. **(E)** Representative photograph for TUNEL staining section of renal tubular epithelial cells (green) in each group. Original magnification: ×400. Scale bar: 20 µm. **(F)** Quantitative evaluation of TUNEL-positive cells in renal tissue. Data displayed are mean ± SEM (*n* > 5). Compared with sham operation group, *p < 0.05. Compared with Control + IR group, #*p* < 0.05.

In order to study the role of AR in ischemia-reperfusion injury, AR was used to interfere with the lentiviral vector to silence the expression of AR in male mice, to observe the changes in renal function and histology after renal ischemia-reperfusion injury, and to detect changes in renal tissue. The effect of apoptosis. Compared with the sham group, the serum creatinine and serum urea nitrogen levels of the IR groups were significantly increased; In the IR group, compared with the Control + IR, the serum creatinine and serum urea nitrogen levels of the AR-shRNA + IR group were significantly increased, while there was no significant difference between the AR-NC group and the Control group (Figures 3A,B). Kidney HE staining showed that many renal tubules were atrophied and dilated in the renal tissues of the IR group, accompanied by vacuoles and edema of epithelial cells, but there were no obvious changes in the renal tissues of the sham groups. In the IR group, the renal tissue damage in the AR-shRNA + IR group was more severe than that of the other two groups (Figure 3C). The renal tissue pathological damage score showed that compared with the Control + sham operation group, the degree of renal tissue damage in each IR group was aggravated. Compared with Control + IR, the degree of renal tissue damage in the AR-shRNA + IR group was aggravated, but there was no significant difference in the degree of renal tissue damage in the NC + IR group (Figure 3D). As can be seen in TUNEL staining, compared with Control + Sham operation group, renal cell apoptosis in each IR group increased; compared with Control + IR, renal cell apoptosis increased in AR + IR group, but no significant difference in NC + IR group (Figures 3E,F). After down-regulating the expression of AR, renal cell apoptosis significantly increased.

### The Effect of Silencing Androgen Receptor Expression on Renal Tubular Cell Apoptosis and miR-21 Expression Level

**FIGURE 4:**
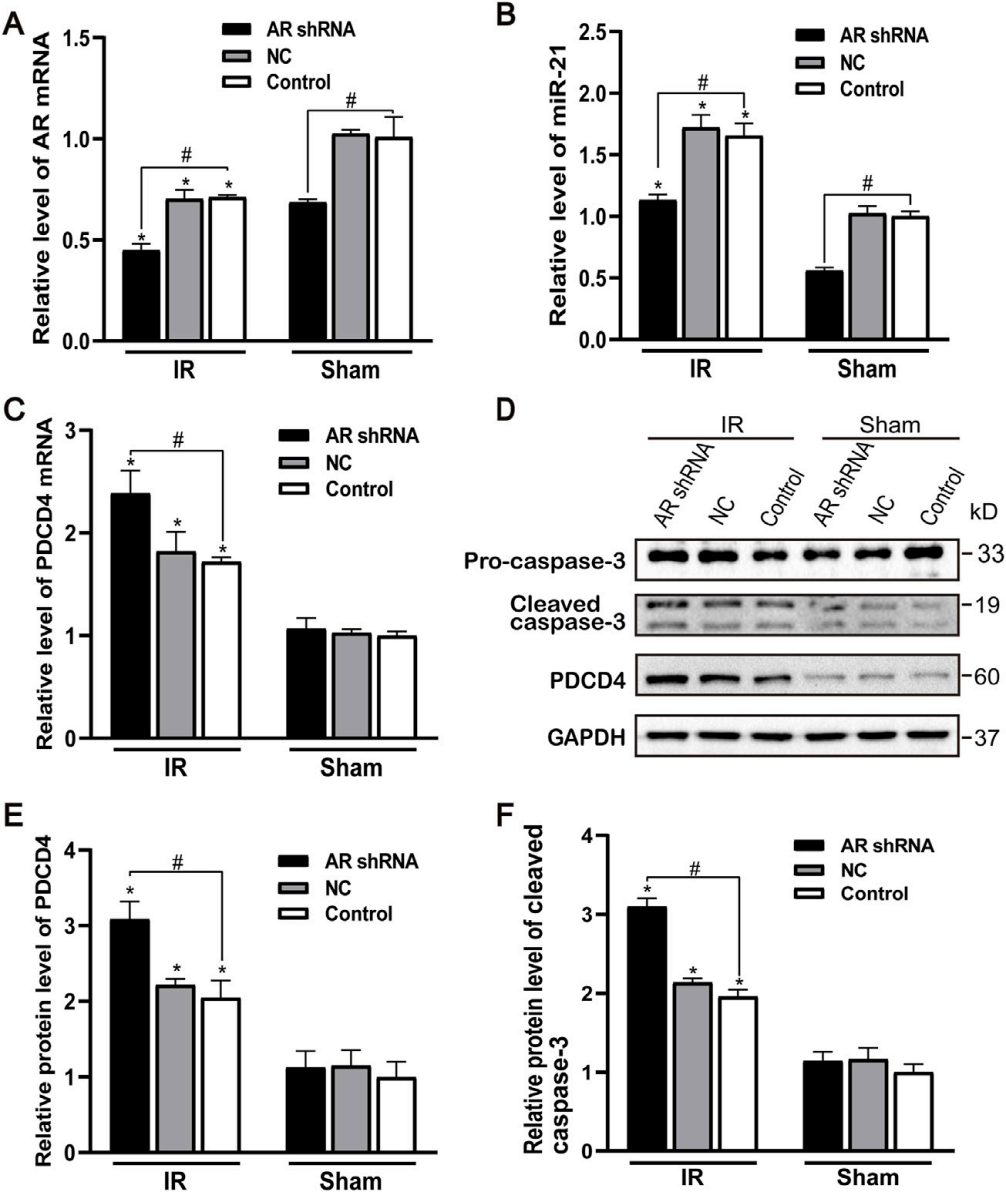
Effect of silencing AR expression on miR-21 level and renal tubular cell apoptosis. **(A)** qPCR showing knocking down AR decreased AR mRNA level after renal ischemia-reperfusion injury in male mice. **(B)** Silencing AR decreased miR-21 level after renal ischemia-reperfusion injury. **(C)** Silencing AR increased PDCD4 mRNA level renal after ischemia-reperfusion injury. **(D)** Western blot showing knocking down AR increased cleaved caspase-3 and PDCD4 protein levels in renal tissue after renal ischemia-reperfusion. **(E)** Quantitative evaluation of PDCD4 protein levels in renal tissue. **(F)** Quantitative evaluation of cleaved caspase-3 protein levels in renal tissue. Data presented are mean ± SEM (*n* > 5). Compared with sham operation group, *p < 0.05. Compared with Control + IR group, #*p* < 0.05.

After silencing the expression of AR in male mice, compared with the control group and the NC group, the AR expression of the AR interference group decreased, and there was no difference between the control group and the NC group. Compared with the sham group, AR expression in each IR group showed different degrees of decline (Figure 4A). After AR interference, the expression of miR-21 in the AR interference group also decreased compared with the control group (Figure 4B). Compared with the sham group, the mRNA level of PDCD4 in each IR group was significantly increased (Figure 4C). We detected and quantified the expression of PDCD4 and caspase-3 protein in kidney tissue and found that the expression of PDCD4 protein was consistent with mRNA. Compared with the Control + sham operation group, the expression of caspase-3 in each IR group increased; compared with the Control + IR group, the expression of caspase-3 protein increased in the AR-shRNA + IR group (Figures 4D–F). These results suggest that apoptosis is more serious after renal ischemia-reperfusion injury, and the down-regulation of AR increases the degree of apoptosis, thereby aggravating renal ischemia-reperfusion injury.

### Effects of AntagomiR-21 and Pre-MicroRNA-21 on the Renal Function and Histological Changes in Mice

**FIGURE 5:**
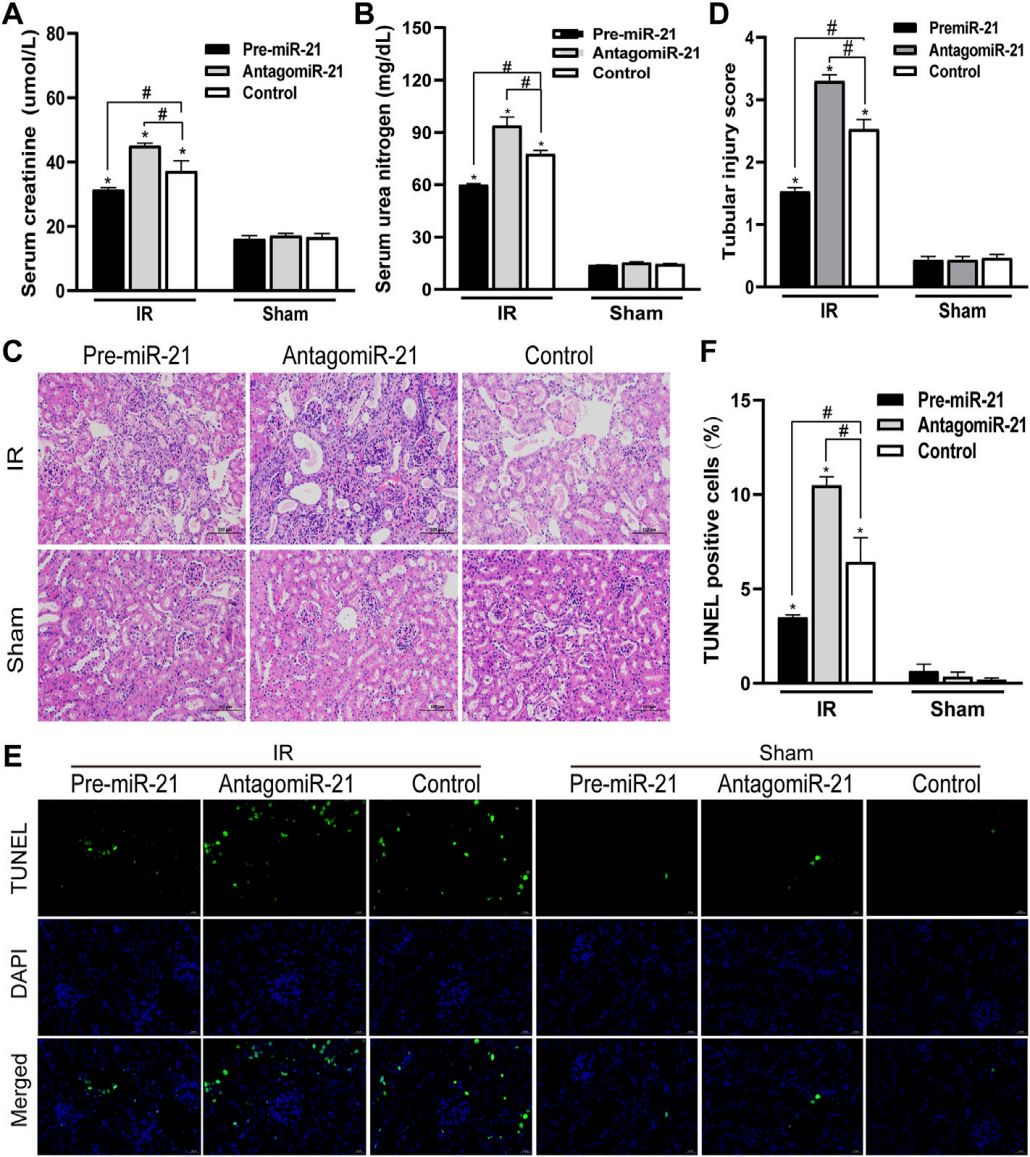
MiR-21 ameliorated the decline of renal function and tubular damage in mice. **(A,B)** pre-miR-21 reduces serum creatinine **(A)** and serum urea nitrogen **(A)** levels after renal ischemia-perfusion, while antagomiR-21 intervention aggravated the decline of renal function. **(C)** HE staining of renal tissue in each group (original magnification ×200). **(D)** Corresponding renal tissue pathological damage score. **(E)** Representative photograph for TUNEL staining section of renal tubular epithelial cells (green) in each group. Original magnification: ×400. Scale bar: 20 µm. **(F)** Quantitative evaluation of TUNEL-positive cells in renal tissue. Data displayed are mean ± SEM (*n* > 5). Compared with sham operation group, *p < 0.05. Compared with Control + IR group, #*p* < 0.05.

In each IR group, compared with the control group, pre-miR-21 significantly inhibited the increase in serum BUN and creatinine levels after IR, while the increase in serum BUN and creatinine levels in the antagomiR-21 + IR group was more significant (Figures 5A,B). Histopathology of the kidney showed that there was no obvious damage and necrosis in the renal tissue of the sham-operated mice. Compared with the Control + IR group, pre-miR-21 treatment can reduce renal tubular atrophy, dilation and swelling, accompanied by a significant reduction in tubular necrosis (Figure 5C). Renal tissue pathological damage score confirms the protective effect of pre-miR-21 on the kidney, while renal tubular injury was not significantly improved in the antagomiR-21 + IR group (Figure 5D). TUNEL staining was used to evaluate the degree of apoptosis of kidney tissue. The results showed that pre-miR-21 pre-treatment can significantly reduce the number of positive cells stained by TUNEL, while the effect of antagomiR-21 is opposite (Figures 5E,F). After ischemia-reperfusion 24 h, cell apoptosis in each IR group was obvious. The supplementation of exogenous miR-21 can effectively reduce renal tubular injury and renal tissue cell apoptosis.

### MicroRNA-21 Reduces Renal Tubular Cell Apoptosis After IR by Targeting PDCD4

**FIGURE 6:**
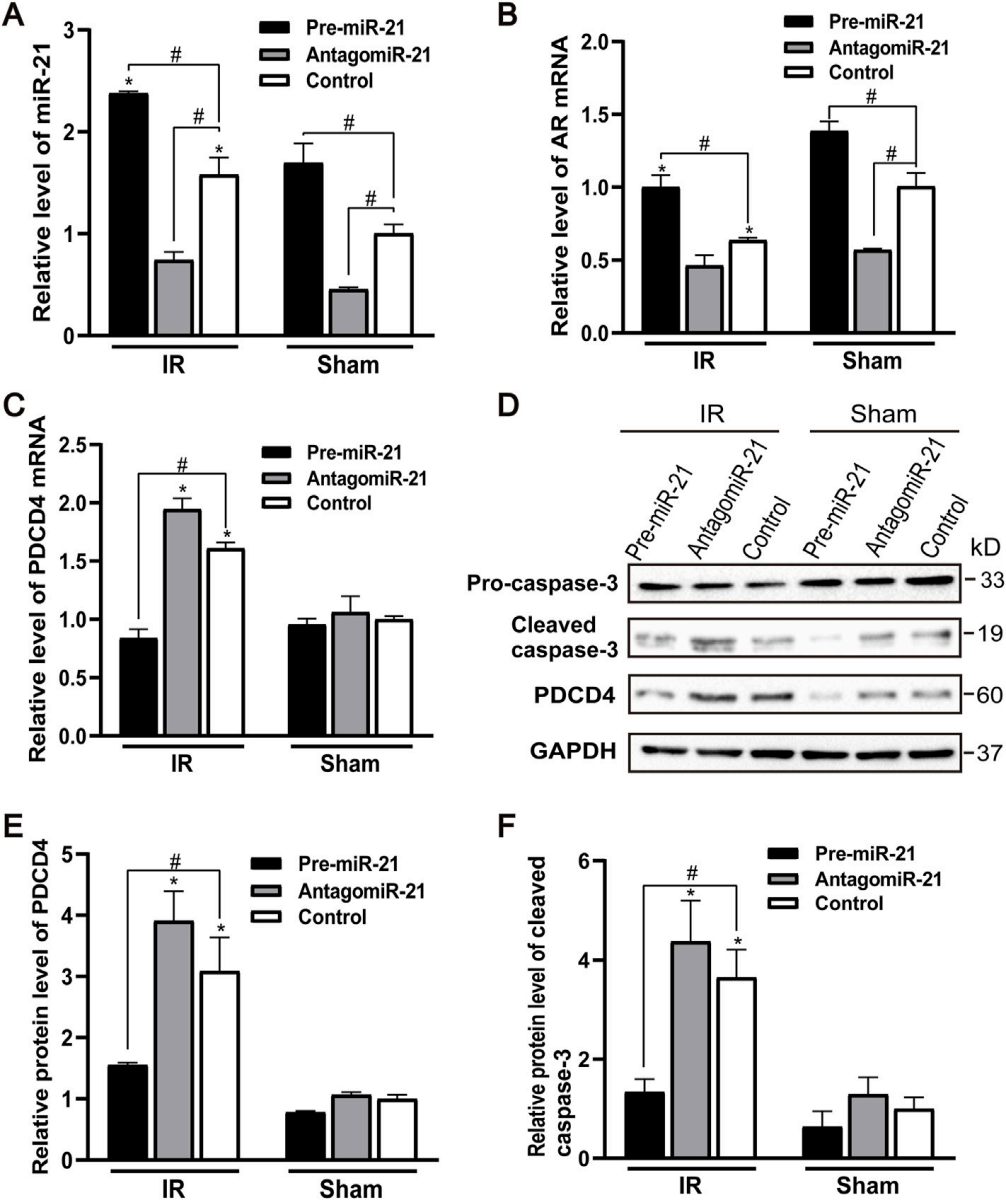
MiR-21 reduces renal tubular cell apoptosis by targeting PDCD4 after ischemia-reperfusion injury. **(A)** qPCR showing pre-miR-21 treatment up-regulated miR-21 level, whereas antagomiR-21 down-regulated the expression of miR-21. **(B)** The expression of AR mRNA in the renal tissue by qPCR. **(C)** PDCD4 mRNA level was negatively correlated with the expression of miR-21 in mice with ischemia-reperfusion injury. **(D)** pre-miR-21 induced the expression of cleaved caspase-3 and PDCD4 protein after renal ischemia-reperfusion, yet antagomiR-21 reduced. **(E)** Quantitative evaluation of PDCD4 protein levels in renal tissue. **(F)** Quantitative evaluation of cleaved caspase-3 protein levels in renal tissue. Data displayed are mean ± SEM (*n* > 5). Compared with sham operation group, *p < 0.05. Compared with Control + IR group, #*p* < 0.05.

The expression of endogenous miR-21 was increased in male mice 24 h after ischemia-reperfusion. Pre-miR-21 treatment up-regulated the level of miR-21, while antagomiR-21 treatment down-regulated the expression of miR-21. However, the expression level of miR-21 did not change significantly after renal IR injury in the antagomiR-21 group (Figure 6A). Pre-miR-21 can increase the expression of AR mRNA, and antagomiR-21 can decrease the expression of AR mRNA (Figure 6B). The expression level of AR in mouse kidney tissue shows the same trend as the expression of miR-21. Compared with the IR group, the PDCD4 mRNA and protein expression levels in the kidney tissue of the pre-miR-21 + IR group mice were reduced, while the PDCD4 mRNA and protein expression levels in the antagomiR-21 + IR mice were increased. The expression level of PDCD4 was negatively correlated with the change of miR-21 expression (Figures 6C–E), which had also been confirmed in other studies (Pan et al., 2019). The expression of pro-apoptotic protein caspase-3 was significantly increased in mice with renal ischemia reperfusion for 24 h. Compared with the control + IR group, pre-miR-21 reduced the expression level of caspase-3, and the up-regulation of miR-21 could alleviate cell apoptosis under the condition of renal ischemia-reperfusion in mice. While the antagomiR-21 + IR group was the opposite, indicating that miR-21 has a protective effect on renal ischemia-reperfusion injury (Figures 6D,F).

## Discussion

Our research results show that there are obvious gender differences in renal ischemia-reperfusion injury in animals. Male mice are more sensitive to IR than female mice and are more susceptible to renal ischemia-reperfusion injury. What mechanism causes gender differences in renal IR? We found that the expression of androgen receptor in the kidney tissues of female and male mice was similar before renal ischemia-reperfusion in the female and male mice with renal ischemia for 45 min and reperfusion for 24 h, but the expression of AR in male mice was obvious after ischemia-reperfusion injury. Decrease, which indicates that the higher expression of AR helps to improve the renal function of female mice after renal ischemia-reperfusion. Our further experiments showed that silencing the expression of AR would aggravate the decline of renal function and renal tubular damage after renal ischemia in mice, and activation of caspase3, a key factor for apoptosis in the Caspase family, was also significantly increased. These results suggest that the down-regulation of AR in animals will increase the degree of apoptosis, thereby aggravating renal ischemia-reperfusion injury. This may be explained by the decline of androgen receptors in the body that makes male mice more sensitive to renal ischemia-reperfusion injury.

A large amount of evidence indicates that hypoandrogenemia is associated with an increased risk of ischemic disease (Yeap, 2018), and androgen is mediated through the transcriptional control of target genes by androgen receptor (AR). Sumiko et al. found in the skeletal muscle ischemia model that the blood flow recovery of male and female AR knockout mice was impaired, apoptosis increased, and the incidence of autologous amputation after ischemia was higher. In *in vitro* studies, AR was silent of vascular endothelial cells showed reduced angiogenesis (Yoshida et al., 2013). Another study showed that the loss of AR in vascular smooth muscle, rather than the loss of AR in endothelial cells, led to ischemia-reperfusion injury in the hind limbs of androgen receptor knockout mice (Wu et al., 2016). After transient middle cerebral artery occlusion in male and female adult rats, a decrease in androgen receptor expression can be detected in the ischemic ipsilateral cerebral hemisphere of male rats (Acaz-Fonseca et al., 2020). These findings suggest the role of androgen receptors in the development of acute ischemic diseases.

MiR-21 is generally up-regulated in several different animal models of kidney disease, as well as human acute kidney injury and chronic kidney disease (Lorenzen, 2015; Loboda et al., 2016; Kölling et al., 2017). From the perspective of miRNA, we analyzed the levels of miR-21 in the kidney tissues of mice of different genders under renal ischemia-reperfusion injury, and found that the expression of miR-21 in the kidney tissues has obvious gender differences. Compared with male mice, the level of miR-21 in female mice increased more significantly after IR injury. The expression of miR-21 in kidney tissue increases after IR, which is considered to be a self-protection mechanism against ischemic injury in animals (Li et al., 2013).

We further analyzed the effect of miR-21 on renal ischemia-reperfusion injury. Up-regulating the expression of miR-21 in male mice by pre-miR-21 treatment can reduce renal IR, while the effect of antagomiR-21 is the opposite. The results of qPCR and Western Blot suggest that miR-21 mainly negatively regulates PDCD4 and reduces the expression level of caspase3, thereby inhibiting renal tubular epithelial cell apoptosis. Programmed cell death 4 (PDCD4) was first thought to be a tumor suppressor gene, which played an anti-tumor effect by promoting apoptosis and inhibiting tumor cell proliferation, invasion, and metastasis (Matsuhashi et al., 2019). As an important downstream target of miR-21, PDCD4 is a pro-apoptotic protein that plays an important role in cell apoptosis. miR-21 directly targets the 3′-UTR region of PDCD4 and down-regulates the expression of PDCD4 to inhibit cell apoptosis (Asangani et al., 2008; Pan et al., 2019).

It has been reported that AR can positively regulate the expression of miR-21 in prostate cancer. On the contrary, AR mRNA level is significantly up-regulated after overexpression of miR-21 using miR-21 mimic, and there is a positive feedback loop between the regulation and expression (Mishra et al., 2014). Similarly, in a study of clear cell renal cell carcinoma, AR enhanced miR-185-5p expression by binding to an androgen response element located on the miR-185-5p promoter, thereby affecting the metastatic pathway, which is dependent on Molecular regulation of AR signaling is also accompanied by sex differences (Huang et al., 2017). Our study preliminarily explored the interaction between AR and miR-21 *in vivo*, the expression level of miR-21 is up-regulated in renal ischemia-reperfusion injury, and silencing AR can reduce the expression of miR-21 and aggravate renal tubular epithelial cell apoptosis. When miR-21 was overexpressed or inhibited, AR mRNA expression showed the same trend of change. These results indicated that AR and miR-21 had a certain synergy in the protection of renal ischemia-reperfusion injury.

## Conclusion

In our experiments, we found that there are obvious gender differences in the sensitivity and tolerance of the kidney to ischemia-reperfusion injury, showing that male mice are more susceptible to renal ischemia-reperfusion injury than female mice. Male mice after ischemia-reperfusion injury can detect a significant decrease in AR expression and an increase in miR-21 level. Silencing the expression of AR can aggravate renal ischemia-reperfusion injury in mice and promote the expression of apoptotic protein caspase3. After *in vivo* application of pre-miR-21 up-regulates endogenous miR-21, it can inhibit the expression of PDCD4 gene and the expression level of apoptotic protein caspase three to play an anti-apoptotic effect, while the effect of antagomiR-21 is opposite. These results prove that protective effect of miR-21 for renal IR is mediated by inhibiting the apoptosis of renal tubular epithelial cells. In the renal ischemia-reperfusion injury model, interference with AR expression will reduce the expression level of miR-21, and up-regulation of miR-21 can promote AR expression. The protective effects of AR and miR-21 on renal ischemia-reperfusion injury have a certain degree of synergy, and there is a feedback loop. Inhibiting this feedback loop will aggravate IR-induced apoptosis of renal tubular epithelial cells. Therefore, AR and miR-21 are expected to become the crucial “keys” for studying the pathogenesis of IR, so as to discover new therapeutic targets for renal IR in the clinic.

## Data Availability

The raw data supporting the conclusions of this article will be made available by the authors, without undue reservation.
